# A calculation method of gas emission zone in a coal mine considering main controlling factors

**DOI:** 10.1038/s41598-021-03090-5

**Published:** 2021-12-08

**Authors:** Yong Chen, Ronghua Liu, Pushi Xuan

**Affiliations:** 1 School of Resource and Environment and Safety Engineering, Hunan University of Science and Technology, Xiangtan, 411201 China; 2 National Key Laboratory of Gas Disaster Detecting, Preventing and Emergency Controlling, Chongqing, 400037 China; 3 China Coal Technology Engineering Group Chongqing Research Institute, Chongqing, 400037 China; 4 Installation Engineering Co., Ltd. of CSCEC 7Th Division, Zhengzhou, 450043 China

**Keywords:** Energy science and technology, Engineering

## Abstract

The gas emission zone is an important parameter for the space–time effect of coal excavation and gas emission. In this paper, according to the effect of roadway excavation, a numerical model of gas emission zone based on the evolution of stress and permeability was established to obtain the width of gas emission zone with different pressure and permeability coefficient. Then the numerical simulation results were verified by measuring the gas content at different depths. Through numerical simulation and field measured data, the theoretical calculation formula is established on the basis of comprehensive consideration of the influencing factors of gas emission zone. The results showed that the gas emission zone increases with the increase of coal seam gas pressure and permeability coefficient when the roadway section and exposure time are the same. The measured gas emission zone, when taking gas content as the index with the same logistic function growth curve, matches the measured results with a relative error of 1.3 to 6%. The validity of the model is also verified by field experiments. The results can provide guidance for mine gas emission and gas drainage design.

## Introduction

Gas is the main threat to safe, efficient mining^1,2^. Determining the width of a gas emission zone after coal excavation allows mine engineers to estimate the intensity of gas emission more accurately; on the other hand, a reasonable sealing depth and length can be determined according to the width of gas emission zone to improve the gas drainage effect^3–5^. Therefore, clearly defining the width of gas emission zone can trace the source of mine gas emission effectively and prevent the occurrence of gas disaster accidents (albeit indirectly).

Many scholars have investigated the gas emission zone in a mine roadway. Galin considered that the shape of the plastic zone around the roadway in the uniform stress field is circular, and the formula for calculating the circular plastic zone was derived^6^. Özgen Karacan considered the geostatistical methods for modeling and prediction of gas amounts and for assessing their associated uncertainty in gas emission zones of longwall mines for methane control^7^. Chen et al. established a plastic zone calculation model considering the creep effect and obtained the characteristics of the change in the plastic zone damage range with time^8^. Zhang et al. studied the width of the gas drainage zone in a coal roadway based on gas emissions^9^. Yang et al. used a numerical simulation method to study the plastic zone range of a roadway under different stress conditions^10^. Zheng et al. investigated the effect of coal damage on permeability and gas drainage performance^11^. Yang et al. analysed the mechanical behaviour of the viscoelastic zone, plastic softened zone, and broken zone around the roadway under conditions involving creep, softening, and expansion of gassy coal. At the same time, establishing a flow-solid coupled model for the gas transportation of the coal around the roadway and analysed the stress state and gas flow around the coal through numerical simulation^12^. Gu et al. studied the relationship between different borehole diameters, the size of the plastic zone, and gas-extraction efficacy^13^. Zhao et al. analysed the influence of the rate of advance of the roadway on the evolution of safe gas drainage channels in fractured overburden^14^.Si et al*.* in order to establish a thorough understanding of gas pressure regimes, and gas emission patterns around a producing multi-level longwall top coal caving face, conducted a suite of in-situ measurements on seam gas pressure, gas composition, and ventilation environment^15^. Additionally, Wang et al*.* analyzed the stress distribution characteristics of roadway surrounding rock damaged zone under non-hydrostatic pressure, and taking the variation of the surrounding rock stress and displacement in circular roadway as the research object, the theoretical calculation model under the condition of non-hydrostatic pressure was established, and the stress and displacement of the surrounding rock damaged zone and plastic zone of circular roadway under non-hydrostatic pressure were given^16^. Yu et al*.* studied the macro–micro mechanical response and energy mechanism of surrounding rock under excavation disturbance, and found that after the roadway excavation, the radial stress and the tangential stress all go through four stages: “pressure relief-recovery-fluctuation-stability”^17^.

Gas emission zone is the main factor affecting the size of gas emission in coal mine, and it is also the basic parameter to determine the length and depth of sealing hole of coal seam during gas drainage. It is difficult to measure the width of gas emission zone in the field. In most cases, historical data are used for reference, which may have certain difference in guiding significance for field work. There are some difficulties in the field measurement of gas emission zone so that it is usually analysed with reference to traditional technical data. During the prediction of mine gas emission, the width of gas emission zone is constant according to different coal qualities and exposure time data. It can be seen that the previous research on the gas emission zone of a coalmine roadway did not consider the calculation method of the roadway and thus cannot better guide engineering practice. The theoretical research into, and practice of mining show that the width of the gas emission zone is not only related to coal quality and coal exposure time, but also related to coal seam gas content, gas pressure, roadway section, permeability coefficient, and other factors. Therefore, it is important to investigate the analysis of gas emission zone sizing to improve the prediction of gas emission and guide the extraction of gas therefrom.

## Numerical simulation of gas emission zone

### Model assumptions

The balance of gas pressure inside the coal body is disturbed, resulting in one-way gas flow during excavation. The intensity of gas emission decreases over time. To obtain the specific range of the gas emission zone of a roadway, some necessary practical assumptions are made to simplify the solution. The basic assumptions are as follows:

(1) The roof and floor of coal seam are gas free and airtight; (2) Coal seam gas contains free and adsorbed gas which can be approximately expressed by a parabolic equation; (3) The gas pressure is evenly distributed in the coal seam and the influence of changes in gas pressure on porosity and permeability is ignored; (4) The gas is regarded as an ideal gas, undergoing laminar flow infiltration in accordance with Darcy’s law; (5) During the process of gas flow, the temperature does not change and obeys the equation of state of an ideal gas.

### Numerical model

The coal seam gas contains free gas and adsorbed gas, and the gas content can be expressed as follows^18^.

1 $$ \begin{aligned} &W = Bnp + \frac{abp}{{1 + bp}}f\left( {\text{t}} \right) \cdot f\left( M \right) \cdot {\text{f}}\left( v \right) \hfill \\ &W = \alpha \sqrt p \hfill \\ \end{aligned} $$

where *B* is the dimensional correction factor; *n* is the porosity of the coal seam, %; *p* is the coal seam gas pressure, MPa; *a* is the adsorption constant of the coal seam, m^3^/t; *b* represents the adsorption constant of the coal seam, MPa^-1^; *f*(*t*), *f* (*M*), and *f* (*v*) are the correction factors for temperature, moisture and fuel; *α* is the coal seam gas content coefficient, m^3^/(m^3^·MPa^1/2^); and *W* is the coal seam gas content, m^3^/t.

The gas is regarded as an ideal gas and its flow in the coal seam is laminar and governed by Darcy’s law.

2 $$ u = - \left( {\frac{{K_{x} }}{\mu }\frac{\partial p}{{\partial x}}i + \frac{{K_{y} }}{\mu }\frac{\partial p}{{\partial x}}j + \frac{{K_{z} }}{\mu }\frac{\partial p}{{\partial z}}k} \right) $$

where *K*_*x*_, *K*_*y*_ and *K*_*z*_ are the coal seam permeability in the *x*, *y*, and *z*-directions; *I*, *j* and *k* are the gas viscosity coefficients.

Under flow, the temperature is constant and the gas density is only related to the gas pressure^19^.

3 $$ \rho { = }\frac{{\rho_{n} }}{{p_{n} }} \cdot p $$

where *ρ* and *ρ*_n_ are the density of gas at pressure *p* and standard atmospheric pressure *p*_n_, kg/m^3^, *p* is the coal seam gas pressure, MPa.

According to the above assumptions and the law of conservation of mass, Eq. (4) can be obtained^20^.

4 $$ \frac{\partial M}{{\partial t}} + \nabla \cdot \left( {\rho u} \right) = 0 $$

where *M* is the density, m^3^/t; *ρ* is the gas density, kg/m^3^; *u* is the average velocity, m/s.

In a homogeneous coal seam where K_x_ = K_y_ = K_z_ = K and $\frac{\partial M}{{\partial t}} = 0$ under steady flow condition, conforming to Laplace’s equation $\nabla^{{2}} p = 0$.

In a homogeneous coal seam:

5 $$ \frac{\partial M}{{\partial t}} + \nabla \cdot \left( {\rho \frac{K}{\mu }\frac{\partial p}{{\partial x}}} \right){ = }0 $$

Under the condition of unidirectional flow, the gas emission from the roadway side is stable and is mainly determined by the coal seam gas pressure and pressure gradient^20,21^, so

6 $$ \begin{aligned} & \lambda { = }\frac{1}{2}\frac{K}{{\mu p_{n} }} \\ & P{ = }p^{2} \\ & q = - \frac{K}{\mu }\frac{p}{{p_{n} }}\frac{dp}{{dx}} = - \frac{K}{{2\mu p_{n} }}\frac{dP}{{dx}} = - \lambda \frac{dP}{{dx}} \\ \end{aligned} $$

where *λ* is the permeability coefficient of the coal seam, m^2^/(MPa^2^·d); *K* is the coal seam permeability, m^2^; *μ* is the dynamic viscosity of the gas, Pa·s; *p*_*n*_ is the normal atmospheric pressure, MPa; *p* is the coal seam gas pressure, MPa; *P* is the square of the gas pressure at a certain position, MPa^2^.

**Figure 1:**
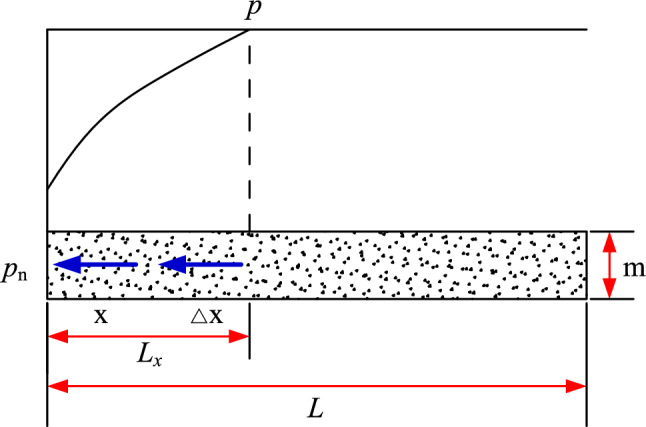
Unidirectional gas flow.

Suppose the thickness of horizontal coal seam is m, the length is *L*, the roadway side gas pressure is P_n_, the original gas pressure in the coal seam is *p*, and the gas flow field length is *L*_*x*_, as the gas continuously migrates from the coal body to the free side of the roadway, the flow field gradually increases in size until it reaches *L*. The gas flow state at any point in the gas flow field changes dynamically with time. The unidirectional gas flow of any roadway section is shown in Fig. 1.

According to the law of conservation of mass, it is in accordance with the flow field of volume △*V* in the gas flow field:

7 $$ \frac{\partial W}{{\partial t}} + \nabla \left[ {\frac{K}{\mu }\left( {\frac{\partial p}{{\partial x}}i + \frac{\partial p}{{\partial y}}j + \frac{\partial p}{{\partial z}}k} \right)} \right] = 0 $$

For any unit area in the flow field, the following conditions are satisfied:

8 $$ \frac{\partial W}{{\partial t}} + \frac{\partial }{\partial x}\left( {\frac{K}{\mu }\frac{dp}{{dx}}} \right) = 0 $$

A second order, non-linear differential equation is thus obtained:

9 $$ \frac{\partial P}{{\partial t}} = \frac{{4\lambda p^{1.50} }}{\alpha } \cdot \frac{{\partial^{2} P}}{{\partial x^{2} }} $$

Equation (9) can be simplified according to the Kriczynski approximation:

10 $$ \begin{aligned} & \frac{\partial P}{{\partial t}} = \frac{{4\lambda p_{1}^{3/4} }}{\alpha } \cdot \frac{{\partial^{2} P}}{{\partial x^{2} }} \\ & P\left| {_{t = 0} } \right. = p_{1} \\ & P\left| {_{x = 0} } \right. = p_{0} \\ & P\left| {_{x = L} } \right. = p_{1} \\ \end{aligned} $$

where *P* is the square of the gas pressure at a certain position, MPa^2^; *t* is the exposure time of the roadway side, d; *λ* is the permeability coefficient of the coal seam, m^2^/(MPa^2^·d); *P* is the square of the gas pressure at a certain position, MPa^2^; *x* is the distance from the roadway side, m; *p*_0_ is the square of the atmospheric pressure, MPa^2^; *p*_1_ is the square of the coal seam gas pressure, MPa^2^; *L* is the limiting length of the gas emission zone in the coal roadway.

Transformation of Eq. (10) by Euler’s equation gives the one-dimensional plane flow field:

11 $$ J[P(x,t)] = \iint {\left\{ {\frac{1}{2}\phi \left( P \right) \cdot \left( {\frac{\partial P}{{\partial x}}} \right)^{2} + \frac{\partial P}{{\partial t}}P} \right\}}dxdt $$

The minimum of $J[P\left( {x,t} \right)]$ must satisfy:

12 $$ \frac{\partial J}{{\partial P_{k} }} = \sum\limits_{e = 1}^{m} {\frac{{\partial J^{e} }}{{\partial P_{k} }}} = 0\quad \left( {k{ = }1,2,3...n} \right) $$

where *P*_k_ is the gas pressure in a given stage; *m* is the total number of units in the region; *n* is the total number of nodes in the same zone.

Simultaneous solution of Eqs. (11) and (12), then taking the partial derivative of the pressure at *n* nodes and setting the partial derivative equal to 0 gives:

13 $$ [K]\left\{ P \right\}_{t} + [N]\left\{ {\frac{\partial P}{{\partial t}}} \right\}_{t} = 0 $$

Solving Eq. (13), we then expand $\left\{ {\frac{\partial P}{{\partial t}}} \right\}_{t}$ in backward difference form:

14 $$ \left\{ {\frac{\partial P}{{\partial t}}} \right\}_{t} = \frac{{\left\{ P \right\}_{t} - \left\{ P \right\}_{t - \Delta t} }}{\Delta t} $$

Substituting Eq. (14) into Eq. (13), we can obtain

15 $$ \left( {[K] + \frac{1}{\Delta t}[N]} \right)\left\{ P \right\}_{t} = \frac{1}{\Delta t}[N]\left\{ P \right\}_{t - \Delta t} $$

If $\left\{ P \right\}_{t - \Delta t}$ in Eq. (14) is a known pressure distribution field, the pressure distribution field $\left\{ P \right\}$ at time *t* can be obtained. Then use $t + \Delta t$ for t in Eq. (14), the pressure distribution field at $t + \Delta t$ time can be obtained.

**Figure 2:**
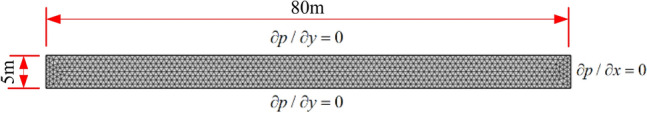
The model of numerical simulation.

The COMSOL solution model is established according to the establishment of numerical model. The thickness of coal seam in the test mine is 3.20–6.64 m, with an average of 4.96 m. Therefore, the numerical model sets the thickness of the coal seam as 5 m, mainly based on the following considerations:(1) The height of the model corresponds to the thickness of coal seam, and the boundary is the roof and floor of coal seam; (2) Coal seam characteristics are mainly divided into horizontal bedding direction and vertical joint direction. According to theoretical research results, coal seam gas emission under the influence of mining is mainly caused by pressure difference seepage between bedding. The section length of the numerical model chosen 80 m mainly because of the permeability coefficient of coal seam is relatively large which up to 0.30m^3^/(m^3^·d), on the other hand, the maximum numerical simulation period of the width of gas emission zone under different pressures and permeability coefficients is 200 d, setting the model length of 80 m can reflect the emission effect of different intervals obviously. The model of numerical simulation is shown in Fig. 2.

### Boundary conditions and parameter configuration

(1) Initial condition

The gas pressure in coal seam at *t* = 0 is usually taken as the initial condition of the flow field.

16 $$ P\left| {_{t = 0} } \right. = p_{1} $$

(2) Boundary conditions

17 $$ \left\{ {\begin{array}{*{20}l} {P\left| {{}_{x = 0} } \right. = p_{0} } \hfill \\ {\frac{\partial P}{{\partial x}}\left| {{}_{x = L} } \right. = 0} \hfill \\ \end{array} } \right. $$

(3) Parameter configuration

**Figure 3:**
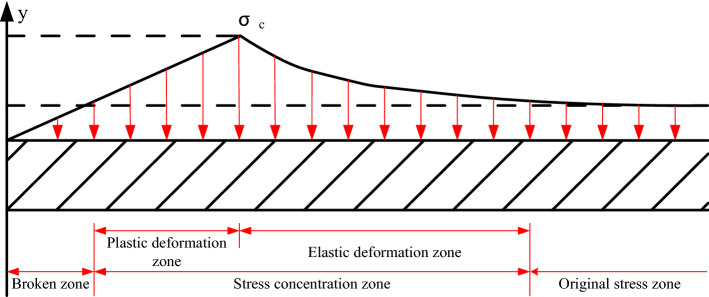
Stress zoning characteristics in the side of a roadway side after coal excavation.

According to different stress states, the coal mass around the roadway can be divided into a seepage open area, seepage directional area, seepage attenuation area, and original seepage area along the radial direction of the roadway. Stress has great influence on coal seam permeability. The permeability of the pressure relief zone can be increased several times even to the extent of hundreds or thousands of times, while the permeability of the stress concentration zone will be weakened so that the gas flow resistance will be increased, and the original stress zone will remain unchanged. Due to the uneven distribution of stress, the gas flow state is not the same at a certain distance into the side of the roadway^11,22,23^, therefore, it is necessary to study the characteristics of stress zoning in the roadway to assess changes in coal seam permeability. Figure 3. shows the broken zone, stress concentration zone and original zone.

Through measuring the volume of drilling cuttings from boreholes with a length of 10–15 m and a diameter of 42 mm, the stress zoning characteristics of roadway side coal body are obtained.The pressure relief zone is 0–4 m, the stress concentration zone is 4–12 m, and the original stress zone is more than 12 m deep. The testing results of drill cutting volume are show in Table1.

**Table 1:** The testing results of drill cutting volume.

Length/m	1	2	3	4	5	6	7	8	9	10	11	12	13	14
Drill cutting mass/kg	1.50	1.40	1.60	2.00	2.30	2.20	2.30	2.20	2.10	1.90	1.90	1.70	1.50	1.70

The vertical initial geostress can be expressed as the product of the overburden rock bulk density and buried depth, and the horizontal geostress is the product of the vertical stress and the correction coefficient used in elastic mechanics theory^24^.

18 $$ \begin{aligned} & \sigma_{v} { = }\gamma H \\ & \mu /\left( {{1 - }\mu } \right) \\ & \sigma_{h} { = }\mu \gamma H/\left( {1-\mu } \right) \\ \end{aligned} $$

where γ is the unit weight of the overlying rock, 2.70 t/m^3^; *H* denotes the burial depth, 315 m; *μ* is the Poisson’s ratio of coal (from the range of 0.10–0.50, we take the middle value of 0.30).

The volume of drilling cuttings and vertical stress in the original stress zone can be considered as fixed values, and the effective stress is expressed as follows.

19 $$ \sigma { = }\sigma_{v} - p $$

According to the relationship betweenthe volume of drilling cuttings, coal seam gas pressure and effective stress, it can be concluded that:

20 $$ \sigma { = }4.006S $$

At the same time, there is a good corresponding relationship between coal seam permeability and stress. Enever et al. studied the relationship between coal seam permeability and stress in Australia and found that the coal seam permeability decreased with the increase of effective stress and changed exponentially^25^.

21 $$ k = k_{0} e^{ - \beta \sigma } $$

where *k* is the permeability under stress, m^2^; *k*_0_ is the initial permeability of coal seam, 0.20 × 10^–15^ m^2^; *σ* is the stress on the coal body, MPa; *β* represents the experimental regression coefficient, 0.05 MPa^−1^.

The change in permeability with depth in the coal body can be obtained by fitting the effective stress and permeability^26^:

22 $$ k = 0.014862 + 0.004965x-0.003584x^{2} + 0.00046086x^{3} -0.0000166x^{4}\quad \left( {R{ = }0.9557} \right) $$

Accordingly:

23 $$ \left\{ {\begin{array}{*{20}l} {\lambda_{1} = -0.000039 \times x^{4}  + 0.0011 \times x^{3} -0.0084 \times x^{2}  + 0.0117 \times x + 0.34893\quad\left( {0 \le x \le 12m} \right)} \hfill \\ {\lambda_{2} = \lambda\quad \left( {x > 12m} \right)} \hfill \\ \end{array} } \right. $$

**Figure 4:**
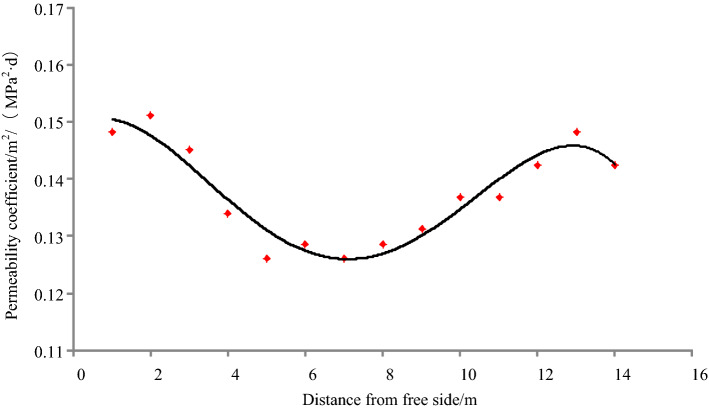
Distribution of permeability in different stress zones after coal excavation.

The distribution of permeability in different stress zones after coal excavation is shown in Fig. 4. The basic parameters of the coal gas are listed in Table 2.

**Table 2:** Gas parameters.

Symbol definition	Parameter	Value	Unit	Symbol definition	Parameter	Value	Unit
λ	Permeability coefficient	0.33	m^2^/(MPa^2^ d)	φ	Porosity	10.90	%
M_ad_	Water content	0.78	%	V_daf_	Volatile matter	7.86	%
A_d_	Ash content	13.91	%	γ	Density of coal	1.56	t/m^3^
a	Adsorption constant	36.76	m^3^/t	b	Adsorption constant	0.9550	MPa^-1^
p	Original gas pressure	1.70	MPa	P_n_	Gas pressure under standard conditions	0.10	MPa

### Analysis of simulation results

**Figure 5:**
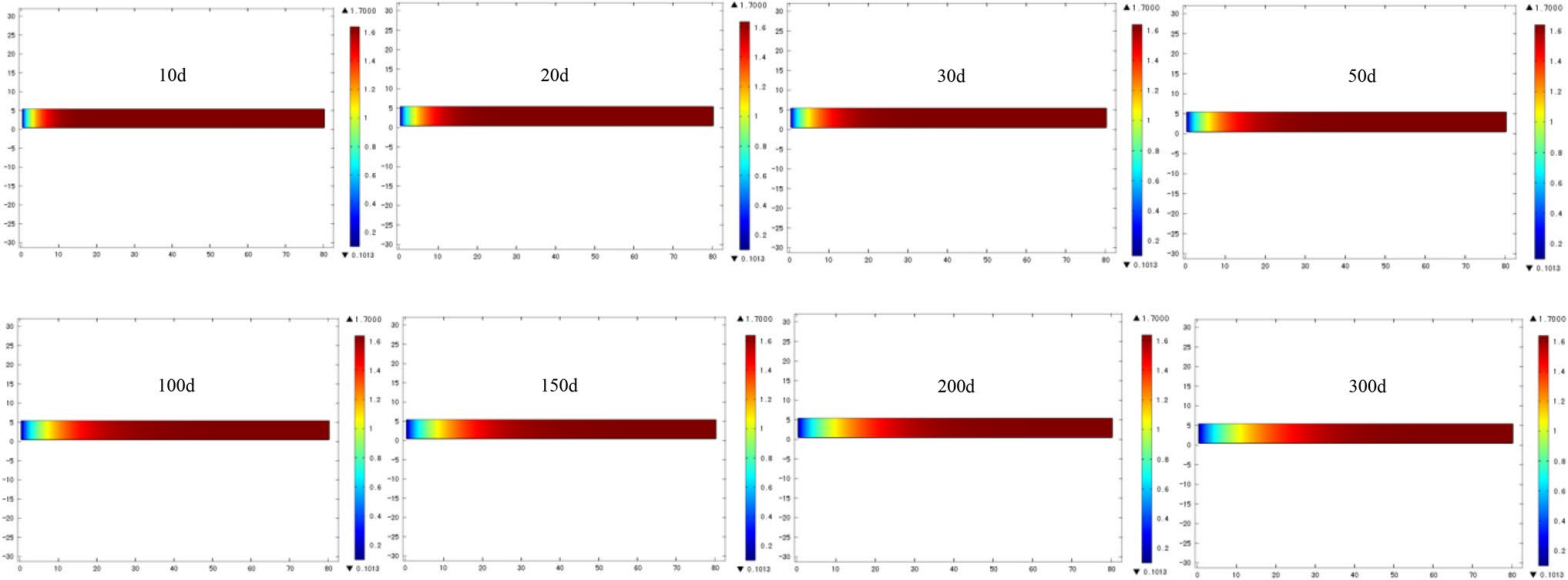
Cloud image of the width of gas emission zone with time distribution under specific parameters.

According to the established numerical model, the variation law of the width of gas emission zone with time under specific parameters was simulated, including 10, 20, 30, 50, 100, 150, 200 and 300 days respectively, as shown in Fig. 5.

It can be found through the simulation cloud image that under the premise of certain parameters such as coal seam gas content, gas pressure and permeability coefficient, the width of gas emission zone shows an increasing trend with time. In order to obtain the distribution law of the width of gas emission zone under different gas pressure and permeability coefficient, further numerical simulation analysis was carried out for the width of gas emission zone under the gas pressure of 1.70 MPa and the permeability coefficient of 0.30 m^2^/MPa^2^ d.

**Figure 6:**
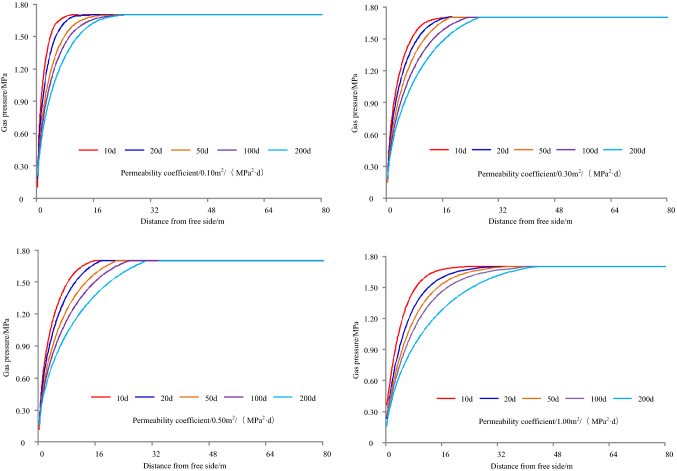
Gas emission zone under different permeability coefficients.

**Figure 7:**
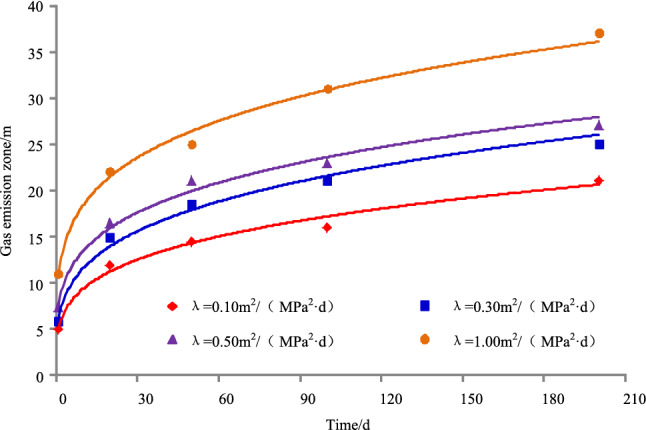
Variation of gas emission zone size with time under different permeability coefficients.

The gas emission zone under different permeability coefficients when the original gas pressure in the coal seam is 1.70 MPa and the cross-sectional area of the roadway is 22.50 m^2^, as shown in Fig. 6. The results of simulation under different permeability coefficients are shown in Table 3, and it can be seen from Table 3 that the variation of gas emission zone size with time under different permeability coefficients, as shown in Fig. 7.

**Table 3:** The results of simulation under different permeability coefficients.

Time/d	Permeability coefficient/m^2^/(MPa^2^ d)	Gas emission zone/m	Permeability coefficient/m^2^/(MPa^2^ d)	Gas emission zone/m	Permeability coefficient/m^2^/(MPa^2^ d)	Gas emission zone/m	Permeability coefficient/m^2^/(MPa^2^ d)	Gas emission zone/m
1	0.10	5	0.30	6	0.50	7.50	1.00	11
20	0.10	12	0.30	15	0.50	16.50	1.00	22
50	0.10	14.50	0.30	18.50	0.50	21	1.00	25
100	0.10	16	0.30	21	0.50	23	1.00	31
200	0.10	21	0.30	25	0.50	27	1.00	37

Taking 1 d as an example, when the permeability coefficient is 0.10, 0.30, 0.50, or 1 m^2^/(MPa^2^ d), the width of gas emission zone is 5 m, 6 m, 7.50 m, or 11 m, respectively. For 20 d, the width of gas emission zone is 12 m, 15 m, 16.50 m, or 22 m, respectively. For 50 d, the width of gas emission zone is 14.50 m, 18.50 m, 21 m, or 25 m, respectively. For 100 d, the width of gas emission zone is 16 m, 21 m, 23 m, or 31 m, respectively. For 200 d, the width of gas emission zone is 21 m, 25 m, 27 m, or 37 m, respectively.

Taking a permeability coefficient 0.10 m^2^/MPa^2^ d as an example, when the exposure time is 1 d, 20 d, 50 d, 100 d, or 200 d, the width of the gas emission zone is 5 m, 12 m, 14.5 m, 16 m, or 21 m, respectively. For 0.30 m^2^/MPa^2^ d, the width of the gas emission zone is 6 m, 15 m, 18.5 m, 21 m, or 25 m, respectively. For 0.50 m^2^/MPa^2^ d, the width of the gas emission zone is 7.50 m, 16.50 m, 21 m, 23 m, or 27 m, respectively. For 1.00 m^2^/MPa^2^ d, the width of the gas emission zone is 11 m, 22 m, 25 m, 31 m, or 37 m, respectively.

With the increase of permeability coefficient and exposure time, the width of gas emission zone increases and gradually tends to be stable when the coal seam gas pressure and roadway section are the same.

**Figure 8:**
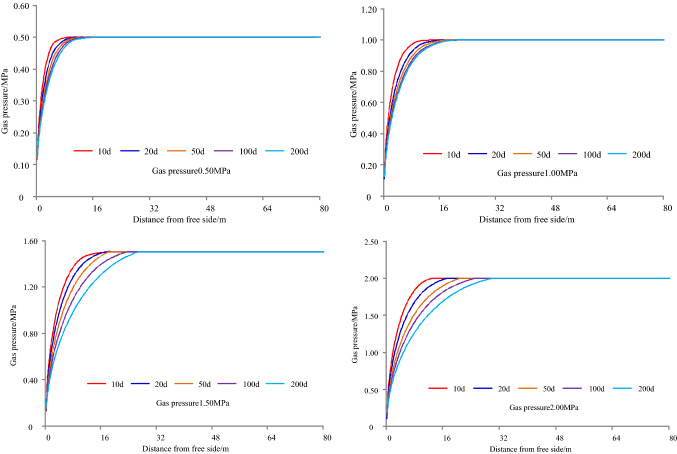
Gas emission zone under different gas pressures.

**Figure 9:**
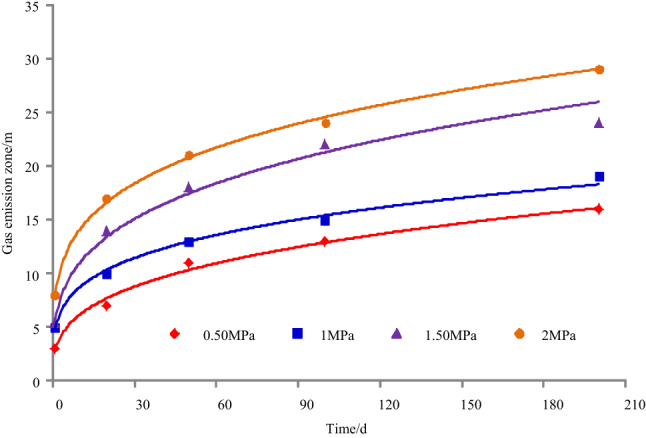
Variation of gas emission zone with time under different gas pressures.

The gas emission zone under different gas pressures when the permeability is 0.30 m^2^/(MPa^2^ d) and the cross-sectional area of roadway is 22.50 m^2^, as shown in Fig. 8. The results of simulation under different gas pressures are shown in Table 4, and it can be seen from Table 4 that the variation of gas emission zone with time under different gas pressures, as shown in Fig. 9.

**Table 4:** The results of simulation under different gas pressures.

Time/d	Gas pressure/MPa	Gas emission zone/m	Gas pressure/MPa	Gas emission zone/m	Gas pressure/MPa	Gas emission zone/m	Gas pressure/MPa	Gas emission zone/m
1	0.50	3	1.00	5	1.50	5.50	2.00	8
20	0.50	7	1.00	10	1.50	14	2.00	17
50	0.50	11	1.00	13	1.50	18	2.00	21
100	0.50	13	1.00	15	1.50	22	2.00	24
200	0.50	16	1.00	19	1.50	24	2.00	29

Taking 1 d as an example, when the gas pressure is 0.50 MPa, 1.00 MPa, 1.50 MPa, or 2.00 MPa, the width of gas emission zone is 3 m, 5 m, 5.50 m, or 8 m, respectively. For 20 d, the width of gas emission zone is 7 m, 10 m, 14 m, or 17 m, respectively. For 50 d, the width of gas emission zone is 11 m, 13 m, 18 m, or 21 m, respectively. For 100 d, the width of gas emission zone is 13 m, 15 m, 22 m, or 24 m, respectively. For 200 d, the width of gas emission zone is 16 m, 19 m, 24 m, or 29 m, respectively.

Taking a gas pressure 0.50 MPa as an example, when the exposure time is 1 d, 20 d, 50 d, 100 d, or 200 d, the width of the gas emission zone is 3 m, 7 m, 11 m, 13 m, or 16 m, respectively. For 1.00 MPa, the width of the gas emission zone is 5 m, 10 m, 13 m, 15 m, or 19 m, respectively. For 1.50 MPa, the width of the gas emission zone is 5.50 m, 14 m, 18 m, 22 m, or 24 m, respectively. For 2.00 MPa, the width of the gas emission zone is 8 m, 17 m, 21 m, 24 m, or 29 m, respectively.

With the increase of gas pressure and exposure time, the width of gas emission zone increases and gradually tends to be stable when the coal seam permeability coefficient and roadway section are the same.

## Field verification of numerical simulation

Coal seam gas content is one of the most direct parameters to determine the width of gas emission zone, which can be determined by testing the gas content of coal seam with different exposure time and different depth. The research results showed that the higher the coal seam gas content and the higher the gas desorption speed. In a certain exposure time, there is a good linear relationship between the gas desorption speed and the coal seam gas content^27,28^.

$$ W = AV_{1} + B. $$

Where *W* is the gas content of coal seam, m^3^/t; *V*_1_ is the gas desorption rate at the first minute after the coal sample is separated from the coal body, m^3^/t.min; *A*, *B* are the constant.

When A and B are known, the gas content of coal seam can be quickly determined by collecting coal samples and measuring the gas desorption rate (*V*_1_).

**Figure 10:**
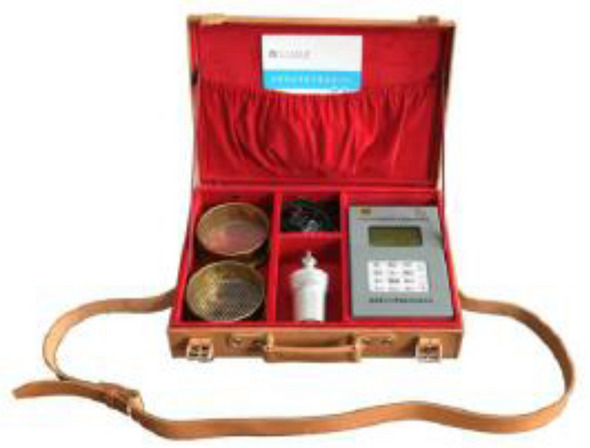
Coal seam gas content measuring device.

**Figure 11:**
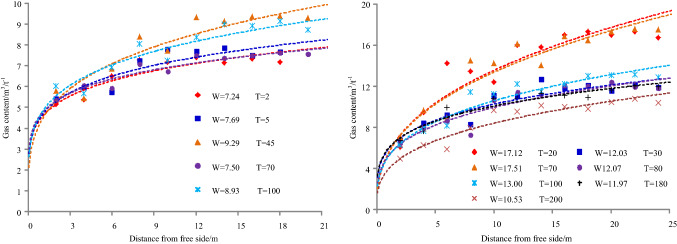
Field measurement of gas emission zone.

To verify the numerical simulation results, the coal seam gas content is selected as the inspection index for field test. In this paper, coal seam gas content measurement instrument using CHP50M coal seam gas content rapid measuring instrument which is based on the coal seam gas content and gas desorption speed linear correlation results, at the same time, using high-precision combined flow sensor through borehole sampling to measure the gas desorption speed of coal sample, so that can achieve the rapid determination of coal seam gas content in 20 min. CHP50M as shown in Fig. 10. The gas content of coal seam is measured at 2 m intervals by borehole drilling to a depth of 20–25 m along the seam. The coal seam mined in the test mine is No.3 coal seam of Shanxi Group, with a thickness of 3.20–6.64 m and an average of 4.96 m. The measured gas contents are shown in Table 5, and it can be seen from Table 5 that the field measurement of gas emission zone, as shown in Fig. 11.

**Table 5:** Measured gas contents.

Time/d	Original gas content/m^3^ t^−1^	Residual gas content/m^3^ t^-1^											
		2 m	4 m	6 m	8 m	10 m	12 m	14 m	16 m	18 m	20 m	22 m	24 m
2	7.24	5.13	5.36	6.48	7.26	7.15	7.38	7.14	7.33	7.17	–	–	–
5	7.69	5.41	5.95	5.72	7.23	7.79	7.67	7.85	7.49	7.66	–	–	–
45	9.29	5.77	5.43	6.84	8.40	7.71	9.32	9.14	9.36	9.38	9.27	–	–
70	7.50	5.32	6.04	5.92	7.06	6.73	7.50	7.35	7.48	7.63	7.55	–	–
100	8.93	6	5.67	6.92	8.03	7.22	8.35	8.97	8.91	9.12	8.70	–	–
20	17.12	6.15	9.45	14.24	13.48	12.39	16.02	15.82	17.01	17.34	16.98	17.36	16.73
30	12.03	6.87	8.45	9.20	8.32	11.13	11.04	12.68	11.85	12.06	11.54	12.17	11.89
70	17.51	7.13	9.67	8.93	14.48	14.27	16.14	14.06	16.87	16.48	17.35	17.61	17.56
80	12.07	6.51	8.01	8.67	7.31	10.67	11.40	11.23	12.11	12.05	12.40	11.98	11.79
100	13	6.32	7.71	8.23	11.4	11.23	12.21	11.52	12.19	12.98	13.04	13.11	12.87
180	11.97	6.78	7.66	9.95	9.66	10.77	10.77	11.27	11.09	10.89	11.65	12.01	11.93
200	10.53	5.03	6.29	5.93	8.05	9.63	9.52	10.10	9.97	9.82	10.43	10.80	10.37

Dai found that according to the measurement results of gas content in coal seams of different depths, the measured value of gas content (*W*) is the Logistic growth function of the distance (*L*) between the deep coal body and the coal wall^29^. Equation (24) can be obtained.

24 $$ W = a \cdot \frac{{W_{0} }}{{1 + b \cdot e^{ - cL} }} \cdot t^{d} $$

where *W* is the gas content of coal seam, m^3^/t; *W*_0_ is the original gas content of coal seam, m^3^/t; *L* is the distance between the coal body and the coal wall, m; t is the exposure time, d; *a*, *b*, *c*, *d* are the constant.

**Figure 12:**
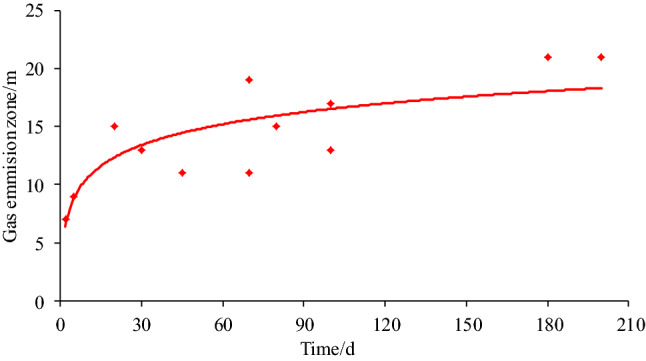
The variation rule of gas emission zone with time.

According to the measurement results of coal seam gas content, the coal seam gas content fluctuates slightly and tends to be stable when it reaches a certain drilling depth. The relationship between coal seam gas content and roadway side distance conforms to the logistic function. When the gas content of coal seam at a certain measuring point is compared with the latter two results, it meets the relationship of $\left| {W_{{\text{n}}} -W_{{n + 1}} } \right|/W_{{\text{n}}} \le 5\%$ and $\left| {W_{{\text{n}}} -W_{{n + 2}} } \right|/W_{{\text{n}}} \le 5\%$, it can be considered that it has entered the original coal zone. The width of gas emission zone determined by use of a logistic growth function is basically consistent with the results of numerical simulation. The width of gas emission zone corresponding to coal seam gas content at different time is shown in Table 6, and the variation rule of gas emission zone with time is shown in Fig. 12.

**Table 6:** The width of gas emission zone corresponding to coal seam gas content at different time.

Time/d	Original gas content/m^3^ t^-1^	Width of gas emission zone/m	Average/m
2	7.24	6–8	7
5	7.69	8–10	9
45	9.29	10–12	11
70	7.50	10–12	11
100	8.93	12–14	13
20	17.12	12–14	13
30	12.03	18–20	19
70	17.51	14–16	15
80	12.07	16–18	17
100	13	20–22	21
180	11.97	20–22	21
200	10.53	14–16	15

## Analysis of the method of calculation of the size of the gas emission zone

### Main controlling factors

In the process of coal seam excavation, the balance state of in-situ stress and gas pressure of coal body is destroyed, and the coal body around the roadway forms pressure relief zone, stress concentration zone and original stress zone from the outside to the inside. Due to the change of coal body stress, the physical characteristics of coal body are changed, and the parameters such as permeability and gas pressure show obvious differences^23,30^. The intensity of gas emission and the form of gas flow field in coal seam are very different in different positions. The fundamental reason is that there are certain differences in gas content, gas pressure, gas permeability and exposure time in different positions. Due to the pressure relief effect of roadway excavation, the gas in the coal body gushes out continuously, and the gas content in the coal body decreases. With the increase of exposure time, the width of gas emission zone also expands from the surface of coal wall to the depth of coal body until the original gas zone of coal body. There is an obvious function relationship between the gas content of coal body and the distance between coal wall and coal body. The gas content increases from the coal wall to the coal body. When the increase approaches to the original gas zone of coal body, the gas content also tends to be stable^31,32^.

The coal seam gas content is not only the source of gas emission but also a decisive factor affecting gas emission intensity. The higher the coal seam gas content, the larger the gas emission and the faster they occur. On the other hand, coal seam gas pressure determines the size of coal seam gas content and gas flow power which is the concrete embodiment of the thermal movement of free gas molecules in the coal. The intensity of gas emission to free space depends on the gas pressure gradient. The larger the emission intensity, the larger the influence range of gas content in the coal body forming the roadway side per unit time^33,34^. The larger the permeability coefficient of coal seam, the smaller the gas flow resistance, the faster the flow and the greater its volume^35^. The section of roadway determines the exposed area of coal body and affects the failure of coal seam, the redistribution of secondary stress and the expansion and development of fracture channels. Gas emission from coal is proportional to the exposed area when under specific conditions. Over time, the gas pressure decreases from the free side to the coal body. The width of the gas emission zone increases with the exposure time and tends to a stable value after a certain amount of aging whereupon it remains stable^36^.

### Calculation model

On the one hand, gas content is the Logistic growth function of the distance between the deep coal body and the coal wall. On the other hand, gas emission from coal roadway heading face mainly includes gas emission from coal wall and gas emission from falling coal in accordance with Chinese National Standards (AQ1018-2006).

For gas emission from coal wall and falling coal can be represented as Eq. (25) and Eq. (26) respectively.

25 $$ q_{1} = D \cdot v \cdot 0.026 \cdot \left( {0.0004 \cdot V_{r}^{2} + 0.16} \right) \cdot W_{0} \cdot \left( {2\sqrt{\frac{L}{v}}  - 1} \right) $$

26 $$ q_{2} = S \cdot v \cdot r \cdot \left( {W_{0} - W_{c} } \right) $$

where *q*_1_ is the gas emission from coal wall, m^3^/min; *D* is the exposed perimeter of roadway coal wall, m; *v* is the average driving speed of roadway, m/min; *V*_*r*_ is the volatiles from coal samples,%; *W*_0_ is the original gas content of coal seam, m^3^/t; *L* is the length of roadway heading,m; *q*_2_ is the gas emission from falling coal, m^3^/min; *S* is the section of heading roadway, m^2^; *r* is the density of coal, t/m^3^; *W*_c_ is the residual gas content of coal seam, m^3^/t.

According to the results of numerical simulation and model verification, the width of the gas emission zone is proportional to the gas content, permeability coefficient, exposure time, and roadway cross-section. According to the previous research results, a hypothetical calculation model is established:

27 $$ L = a_{1} \cdot W^{{b_{1} }} \cdot \lambda^{{e_{1} }} \cdot t^{{c_{1} }} \cdot S^{{d_{1} }} $$

where *L* is the width of the gas emission zone, m; *W* is the coal seam gas content, m^3^/t; *λ* is the permeability coefficient, m^2^/(MPa^2^ d); *t* is the time, d; *S* is the roadway cross-sectional area, m^2^; a_1_, b_1_, c_1_, d_1_, and e_1_ are constants.

The formula of width of gas emission zone can be obtained through the maximum inheritance method used in data fitting and data-merging using mathematical software:

28 $$ L = 1.371 \cdot W^{0.663} \cdot \lambda^{0.280} \cdot t^{0.232} \cdot S^{0.042} \left( {R{ = }0.9843} \right) $$

The chi-squared coefficient of the model is 2.0171 and the hypothesis test statistical variable *F* is 930.988 so the convergence criterion is satisfied.

### Verification of calculation method

To test the applicability and accuracy of the calculation method of gas emission zone, field measurements are adopted, and the corresponding difference value was analyzed with the calculated results. The calculated error in gas emission zone size at different positions are shown in Table 7.

**Table 7:** Calculated error in gas emission zone size at different positions.

Coal seam gas content/m^3^/t	Time/d	Cross-sectional area/m^2^	Permeability coefficient m^2^/(MPa^2^ d)	Calculated value/m	Measured value/m	Relative error %
8.72	50	12.15	0.333	11.66	11	6.00
9.17	15	12.15	0.333	9.12	9	1.30
9.54	10	12.15	0.333	8.52	9	5.30
15.28	100	22.50	0.333	20.38	21	3.00
16.33	150	22.50	0.333	23.40	23	1.70

The verification results showed that with the increase of coal seam gas content, exposure time, and roadway section, the calculated and measured results of gas emission zone show an increasing trend. The relative error between the calculated and measured results of gas emission zone is between 1.30 and 6.00% under different coal seam gas contents, exposure times, and roadway sections. The calculation method of gas emission zone size can thus be deemed accurate.

**Figure 13:**
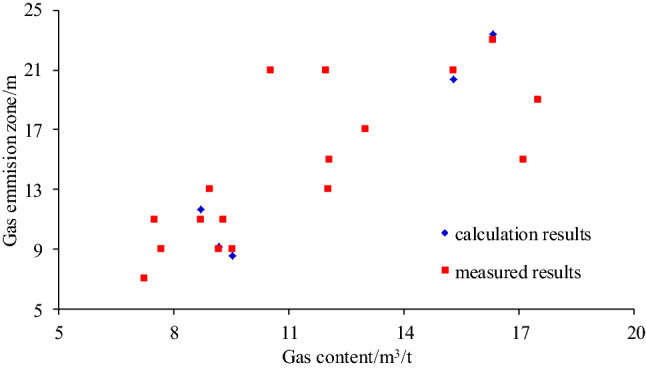
The calculated and measured results of gas emission zone.

According to the comparative analysis between the measured results and the numerical simulation results, the width of the gas emission zone obtained by the calculation method is basically the same as the width of the gas emission zone measured by the coal seam gas content as the index, as shown in Fig. 13.

Chen carried out a study of the plastic zone range of a roadway considering the creep effect showed that the plastic zone of a roadway will increase with time due to the creep effect and finally approach a certain value. After the theoretical calculation, the gas pressure and gas content of coal seam are measured and verified on the spot. The final gas pressures of boreholes are 2.71 MPa, 2.54 MPa, 2.30 MPa and 2.02 MPa, respectively. According to the relationship between gas pressure and gas content, the calculated gas content of coal seam are 14.98 m^3^/t, 14.63 m^3^/t, 14.09 m^3^/t and 13.36 m^3^/t, respectively. The measured values of gas content are 14.12 m^3^/t, 13.92 m^3^/t, 13.68 m^3^/t and 12.49 m^3^/t, respectively. It can be seen that the calculated gas content consistent with the measured gas content with a maximum phase difference of only 0.86 m^3^/t^8^. There is a good consistency between the existing research and the research results of this paper.

Scholars at home and abroad have carried out a lot of research on gas emission zone. Özgen Karacan carried a studied on modeling and prediction of ventilation methane emissions of U.S. longwall mines using supervised artificial neural networks^37^. Mucho found that methane emissions can originate from three major sources during longwall mining in which including gas emissions from the ribs surrounding the bleeder ventilation system^38^. Zhang through establish the physical and mathematical model of coal wall gas emission to deduce the calculation formula of coal lane gas emission zone, so as to study the change rule of coal lane gas emission zone width with time^9^. Sun established the mathematical model of the unidirectional unstable flow of coal roadway gas in the finite flow field and carried out numerical calculation, and then deduced the calculation formula of the width of the coal roadway gas pressure relief discharge zone^39^. Wang obtained analytical expressions of coal stress and volumetric strain through elastoplastic mechanical model of coal body strain softening and dilatation around roadway. At the same time, the gas migration coupling model of roadway pressure relief and matrix shrinkage effect was established by taking permeability as a bridge, and the gas migration law and influencing factors of coal body around roadway were obtained, and the equivalent width of roadway pre-drainage gas under different conditions was determined^40^.

In this paper, according to the effect of roadway excavation, a numerical model of gas emission zone based on the evolution of stress and permeability was established to obtain the width of gas emission zone with different pressure and permeability coefficient. Then the numerical simulation results were verified by measuring the gas content at different depths. Through numerical simulation and field measured data, the theoretical calculation formula is established on the basis of comprehensive consideration of the influencing factors of gas emission zone. The results showed that the gas emission zone increases with the increase of coal seam gas pressure and permeability coefficient when the roadway section and exposure time are the same. The validity of the model is also verified by field experiments. Through the comparative analysis with the previous research results, we can found that the research methods and main controlling factors in this paper are different from previous studies, but there is a good consistency between the existing research and the research results of this paper. The results can provide guidance for mine gas emission and gas drainage design.

## Main conclusions

A gas emission zone model considering the main controlling factors is proposed. The model has good applicability and can be used to calculate the gas emission zone extent under different gas pressures, gas contents, permeability coefficients, and roadway sizes.

With the increase of coal seam gas content, exposure time, and roadway cross-section, the calculated and measured extents of the gas emission zone show an increasing trend. The increase in size of the gas emission zone with time basically conforms to the growth trend of a logistic function. The method of calculation of gas emission zone size considering the main controlling factors is established based on the numerical simulation and field measurement. The coal seam gas content is taken as the index to verify the model and the measurement results verified the validity of the gas emission zone calculation model to good effect.

Four main controlling factors are considered in the calculation of gas emission zone size. In the future, more influencing factors will be further considered and the calculation methods of coal with different degrees of metamorphism will be studied.
